# Bioinspired photocatalytic systems towards compartmentalized artificial photosynthesis

**DOI:** 10.1038/s42004-023-01069-z

**Published:** 2023-12-04

**Authors:** Laura Velasco-Garcia, Carla Casadevall

**Affiliations:** 1 Institute of Chemical Research of Catalonia (ICIQ), The Barcelona Institute of Science and Technology, Avinguda dels Països Catalans, 16, 43007 Tarragona, Spain; 2 Department of Physical and Inorganic Chemistry, University Rovira i Virgili (URV), C/ Marcel.lí Domingo, 1, 43007 Tarragona, Spain

**Keywords:** Photocatalysis, Self-assembly, Synthetic chemistry methodology, Artificial photosynthesis

## Abstract

Artificial photosynthesis aims to produce fuels and chemicals from simple building blocks (i.e. water and carbon dioxide) using sunlight as energy source. Achieving effective photocatalytic systems necessitates a comprehensive understanding of the underlying mechanisms and factors that control the reactivity. This review underscores the growing interest in utilizing bioinspired artificial vesicles to develop compartmentalized photocatalytic systems. Herein, we summarize different scaffolds employed to develop artificial vesicles, and discuss recent examples where such systems are used to study pivotal processes of artificial photosynthesis, including light harvesting, charge transfer, and fuel production. These systems offer valuable lessons regarding the appropriate choice of membrane scaffolds, reaction partners and spatial arrangement to enhance photocatalytic activity, selectivity and efficiency. These studies highlight the pivotal role of the membrane to increase the stability of the immobilized reaction partners, generate a suitable local environment, and force proximity between electron donor and acceptor molecules (or catalysts and photosensitizers) to increase electron transfer rates. Overall, these findings pave the way for further development of bioinspired photocatalytic systems for compartmentalized artificial photosynthesis.

## Abstract (web summary)

Artificial photosynthesis aims to produce fuels and chemicals from simple, abundant building blocks, such as water and carbon dioxide, with sunlight as a source of energy. Here, the authors review recent developments in biomimetic, compartmentalized vesicular systems towards artificial photosynthesis, and highlight challenges and opportunities in mimicking this complex natural reaction system.

## Introduction

**Fig. 1:**
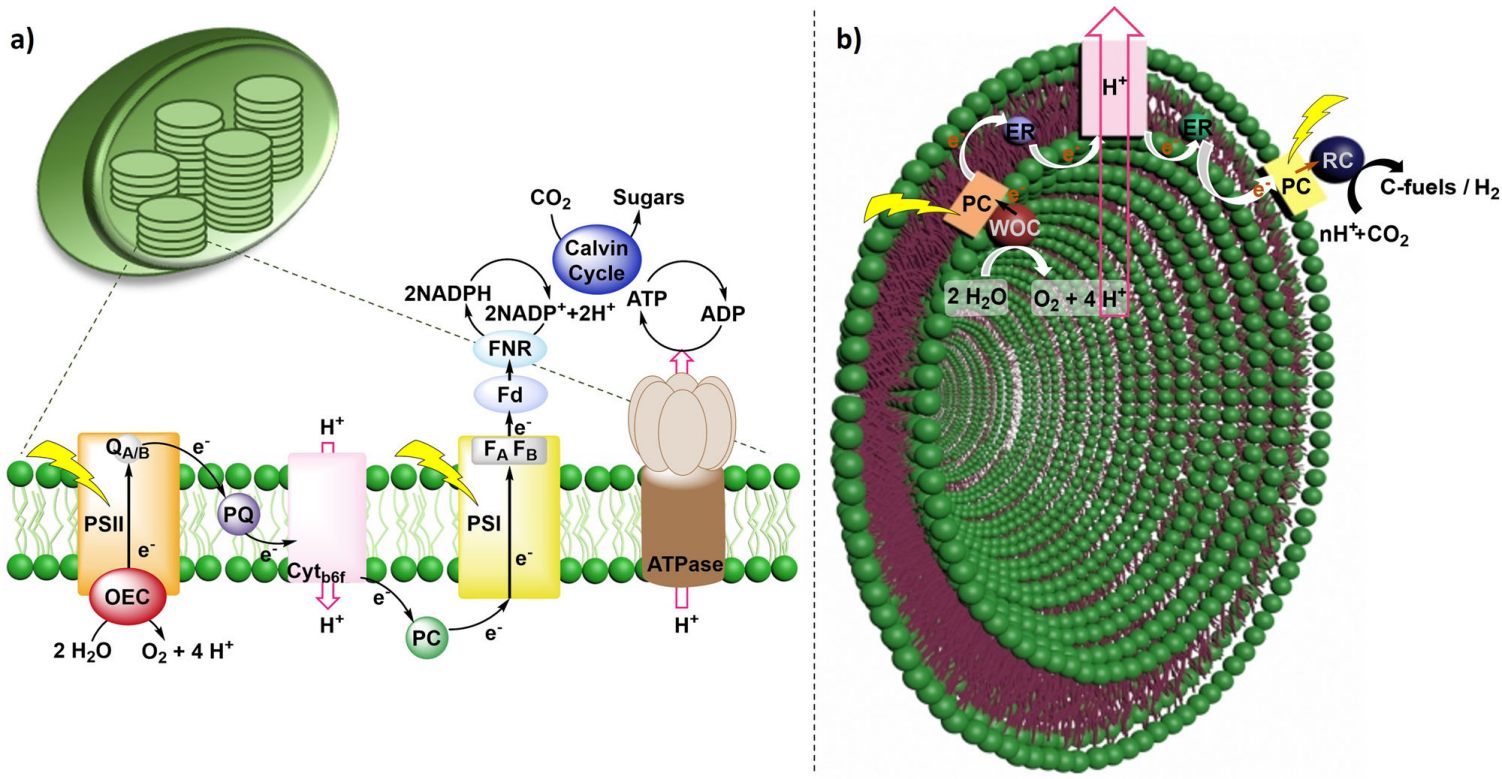
Simplified representation of a thylakoid membrane and an artificial vesicle for compartmentalized AP. **a** Schematic representation of a chloroplast and the zoom of the thylakoid membrane with the most important reaction centres of NP, (**b**) simplified illustration of a synthetic vesicular system and its components to produce solar fuels and chemicals.

Natural photosynthesis (NP) is one of the most important processes that sustains most life forms on Earth. During this process, photosynthetic systems (algae, plants and cyanobacteria) store and convert solar energy into chemical energy in the form of glucose and produce dioxygen as byproduct, by using the simplest available feedstock -water and carbon dioxide- and sunlight as driving force. This process takes place at the thylakoid membrane of chloroplasts, which facilitates the specific spatial arrangement of the different chromophores, reaction centres and mediators, and segregation of the redox reactions that intervene in this biological process (Fig. 1, *left*). The separation of the light-dependent water oxidation (WO) reaction from the generation of reductive equivalents -NADPH and ATP- and subsequent CO_2_ fixation in the dark reactions at each side of the membrane, reduces wasteful back reactions between the oxidation and reduction sites^1–6^. Indeed, the importance of natural membranes extends beyond NP, as lipid bilayers play a pivotal role in biological processes, governing the precise regulation of chemical reactions by creating specialized compartments with distinct conditions and microenvironments throughout biological systems. For instance, the higher degree of compartmentalization of living cells allows for the succession of opposing bioreactions to take place at the same time guaranteeing their function. Therefore, it is becoming progressively evident that a profound and fundamental comprehension of these principles holds significant importance in the context of both chemical reactions and solar energy conversion^7–17^.

In this context, fundamental understanding of the thylakoid special arrangement of light-harvesters and catalytic protein complexes during NP is crucial to mimic this elegant process artificially. Despite (photo)electrochemical systems for artificial photosynthesis (AP) are compartmentalized *per ser* due to the presence of a membrane and two compartments in (photo)electrochemical cells, photocatalytic AP systems rarely take this compartmentalization into account. AP is a forefront technology that aims to produce renewable fuels and chemicals using sunlight and readily available and abundant feedstock such as H_2_O, and CO_2_, mimicking NP but with higher efficiencies and variety of products. Yet, important challenges in AP are (i) charge recombination, (ii) the efficient use of the electrons derived from WO to reduce CO_2_ to C-fuels and chemicals, and (iii) going beyond the simplest products derived from CO_2_ reduction (CO_2_R); which could be potentially solved by the spatial separation of the redox reactions and generation of microenvironments^9,10,18–20^. Therefore, a potential solution to solve these challenges is to develop AP systems that mimic the natural structural arrangement and orchestration of catalysts and photocatalysts in confined spaces separated by a membrane (Fig. 1, *right*). Mimicking this structural feature is crucial for developing efficient catalytic systems for solar fuel and chemical production^9,10,21^. However, achieving this is very challenging due to the different processes and components that need to be considered: (i) choice of the membrane material and its properties, (ii) choice of catalysts, photosensitizers and mediators, (iii) separation of reaction centres, (iv) efficient charge separation, (v) unidirectional electron, proton and energy transfer across the membrane, (vi) product selectivity, among others^9,10^. Additionally, mimicking the generation of proton gradients as an energy source is very important.

This review provides an overview of the recent developments of bioinspired artificial vesicles towards compartmentalized artificial photosynthesis. We compare the different scaffolds used to construct vesicles to mimic the thylakoid membrane to develop photocatalytic systems for fuel and chemical production. These systems are mostly based on lipid-, phospholipid-, polymer- and protein-based bilayer membranes. Moreover, we discuss the recent examples where critical artificial photosynthetic processes, including light absorption, charge transfer, and fuel generation (WO, hydrogen evolution reaction (HER), CO_2_R and overall water splitting (WS) systems), have been investigated. These systems offer valuable insights into enhancing photocatalytic activity and efficiency upon confinement of reaction components within a membrane. From the reported studies we can learn all the factors that need to be considered to assemble photocatalytic artificial vesicles, such as the choice of the membrane material, or the adequate selection and concentration of catalyst, photosensitizers and electron relays to guarantee a unidirectional electron flow. Therefore, paving the way for the development of bioinspired photocatalytic systems for compartmentalized AP. While artificial vesicles may not be used for large-scale fuel production, they serve as platforms to explore the coupling of oxidation and reduction half-reactions within a single system, while providing innovative solutions to tackle charge recombination and cross reactivity of photocatalytic systems in solution, a primary challenge in photocatalytic AP.

It is worth mentioning that in this work we focus only on bilayer membrane vesicular systems. The development of bioinspired confined systems based on metal organic frameworks (MOFs)^22,23^, covalent organic frameworks (COFs)^24–31^, porous materials^32,33^, and other supramolecular assemblies^33–39^ has been reviewed elsewhere and remains out of the scope of this review.

## Common vesicle-based scaffolds used as biomimetic microenvironments

Inspired by nature, in the last years there has been an increasing interest in using artificial vesicles and bilayer-membrane separated compartments to understand better and mimic nature’s compartmentalization of cells. For instance, the light-induced formation of electrochemical gradients across membranes is the crucial natural strategy of cells to harness solar energy and power cellular functions. Those systems are especially promising in the field of synthetic biology since they can be used to construct artificial cells to dissect the building blocks of a living system and the origin of life^7,8,11,40–52^, but also in biosensing^43,53^, and pharmaceutical-medicinal chemistry applications^7,13,43,54–57^. Moreover, in the recent years artificial vesicles have gained increasing interest to study the encapsulation of different biologic or synthetic catalysts and separate reactivity, with the potential to create very complex catalytic systems with synchronised reactivity towards chemical production but also in the field of energy^7–17^. Indeed, those artificial systems can be used as simplified model systems to study different processes in the membrane and at interfaces, such as vectorial electron transport, utilization of proton gradients as source of energy and transport of electrons, energy vectors and small molecules across membranes, relevant processes for AP^9,10,16,17,42,46,49,58–62^.

**Fig. 2:**
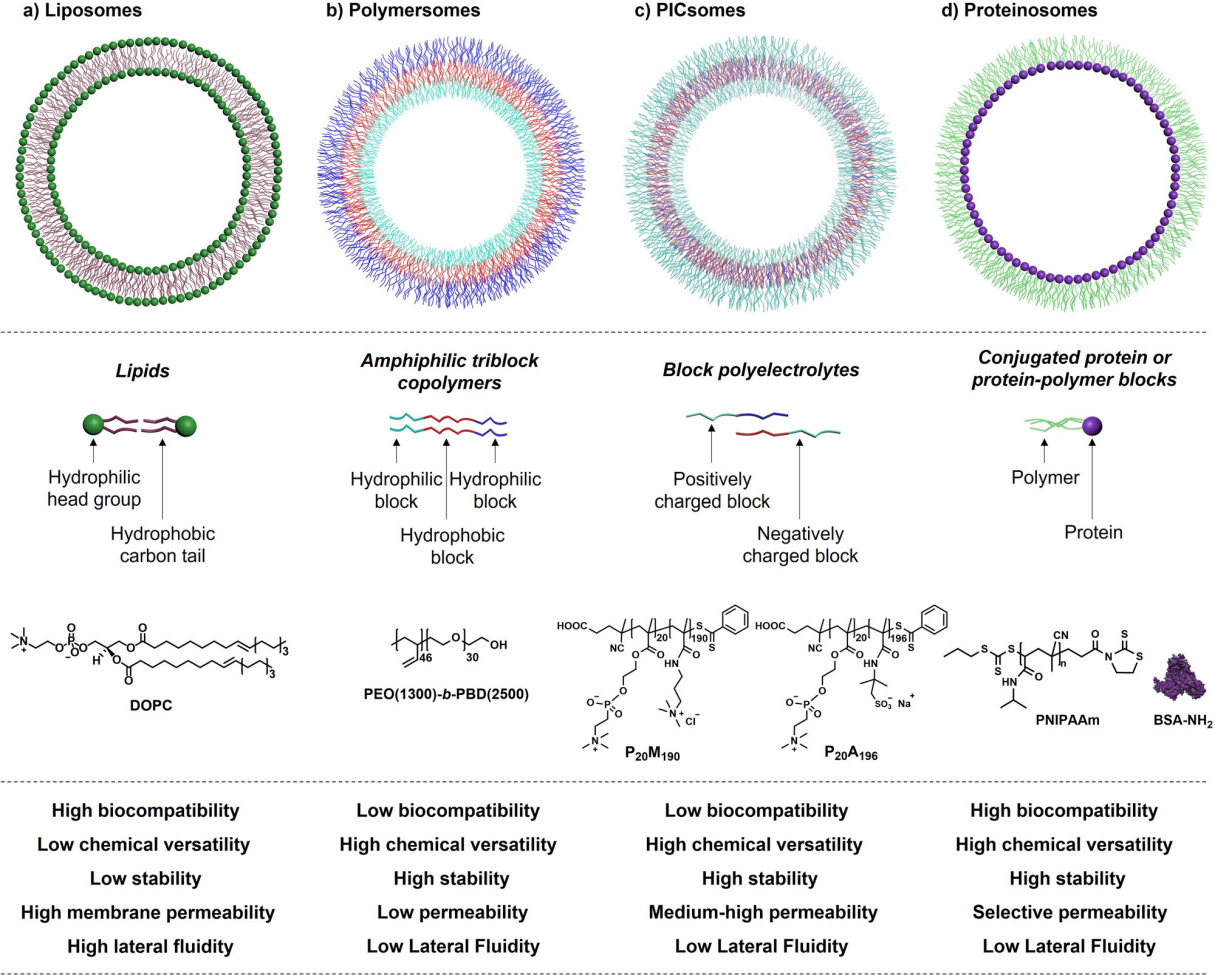
Comparison of the main scaffolds to construct artificial vesicles. Schematic representation of (**a**) liposomes, (**b**) polymersomes, (**c**) PICsomes and (**d**) proteinosomes, together with a simplified scheme of their respective building blocks, and example of common moieties used to build the vesicles, and their main properties, advantages and disadvantages.

Due to their similarity to their natural counterpart, lipids are amongst the most used molecules to generate artificial vesicles^42,63^. Indeed, in the last years within the field of AP most examples rely on the use of liposomes. Nevertheless, other suitable scaffolds have been used to synthesize artificial vesicles for compartmentalized chemical transformations, such as those based on polymer- or protein membranes^42^. It is worth mentioning that the scaffold of choice will directly affect the properties and characteristics of the bilayer membrane. Therefore, its choice is very important for a target application^10,18,52,64,65^. Common scaffolds with their main features, advantages and disadvantages are as follows (Fig. 2):

Liposomes are a type of vesicles formed by the supramolecular self-assembly of amphipathic lipids in an aqueous solution forming a lipid bilayer with a hollow core. These molecules interact with water through polar interactions for their heads and unfavourable interactions for their hydrophobic tails, leading to the formation of bilayer membranes. Since lipids are amphiphilic molecules, they are also surfactants, and thus at low concentrations in water they concentrate at interfaces and lowering the surface tension, with aggregation starting beyond a critical concentration called the critical micelle concentration (CMC). Due to their intrinsic similarity to natural bilayers and biocompatibility, these vesicles are amongst the most commonly used to develop and study biomimetic microcompartments^9,10,51,52,66^. However, liposomes suffer from poor structural stability, limited chemical tunability, can exchange lipid chains between vesicles in solution and show high diffusion and permeability of the membrane, which limits their further applicability as scaffolds to develop fully light-driven WO-to-CO_2_R AP systems^67^.

Polymersomes are vesicles formed by the self-assembly of amphiphilic block copolymers. While exhibiting structural and functional similarities to natural membranes and liposomes, the polymeric bilayer has several advantages over its lipid-based counterpart, such as enhanced mechanical stability, much higher chemical versatility, membrane robustness, and membrane permeability response upon stimuli^42,51,56,68–71^. Moreover, their membrane can be modified to make them more biocompatible and responsive to pH changes or light stimuli, among others^72–79^. Therefore, in the recent years, polymersomes have been used to develop artificial compartmentalized systems for various applications, including drug delivery, biomedical applications, biosensing, chemical biology and catalysis^52,56,67,80–84^. More recently, polymersomes are gaining increasing interest in the field of energy and chemical conversion in AP^67^. Even so, the field is in its infancy and the examples of polymersomes for AP-related applications are very limited^9,67^.

PICsomes are polyion complex vesicles that are self-assembled structures formed from oppositely charged block polyelectrolytes, which are polymers containing ionic or ionizable groups^52,85^. These structures consist of a bilayer membrane composed of these polyelectrolytes, where the electrostatic interactions between positively and negatively charged blocks drive the assembly. The resulting vesicles have a semipermeable membrane that allows for the controlled transport of substances across the bilayer. Moreover, the resulting properties of the assembly depend on the characteristics of the block polymers, such as their polydispersity, charge, block ratio, length and molecular weight. PICsomes have garnered interest for various applications, including drug delivery and nanoreactors, due to their unique properties and the ability to tailor their structure and functionality^71,78,85–90^.

Proteinosomes are vesicles constructed by a monolayer of conjugated proteins or protein-polymer moieties as building blocks with a hydrophilic lumen. Their compositions render the membrane with chemical versatility, selective permeability and high encapsulation efficiency^52,91,92^. Those properties make them highly used in the field of chemical biology to construct proteinosome-based protocells^52,85^.

Recently, the interest in mimicking nature’s complex synchronization has resulted in several studies using artificial vesicles to study compartmentalized energy transfer and conversion processes. However, among the studies reported so far in the field of AP, in most cases the reported examples are only able to perform one transformation, such as light harvesting, proton transfer across membranes, or one of the redox half-reactions such as WO, HER or CO_2_R, but using non-productive sacrificial reagents, such as triethylamine for HER or sodium persulfate for WO. Still, the coupling of both half-reactions (oxidation and reduction) to produce whole-cell photocatalytic systems for WO-to- CO_2_R or WO-to-HER has been very challenging and remains scarce^9,10,93^.

## Artificial systems for electron, proton and energy transfer across membranes

**Fig. 3:**
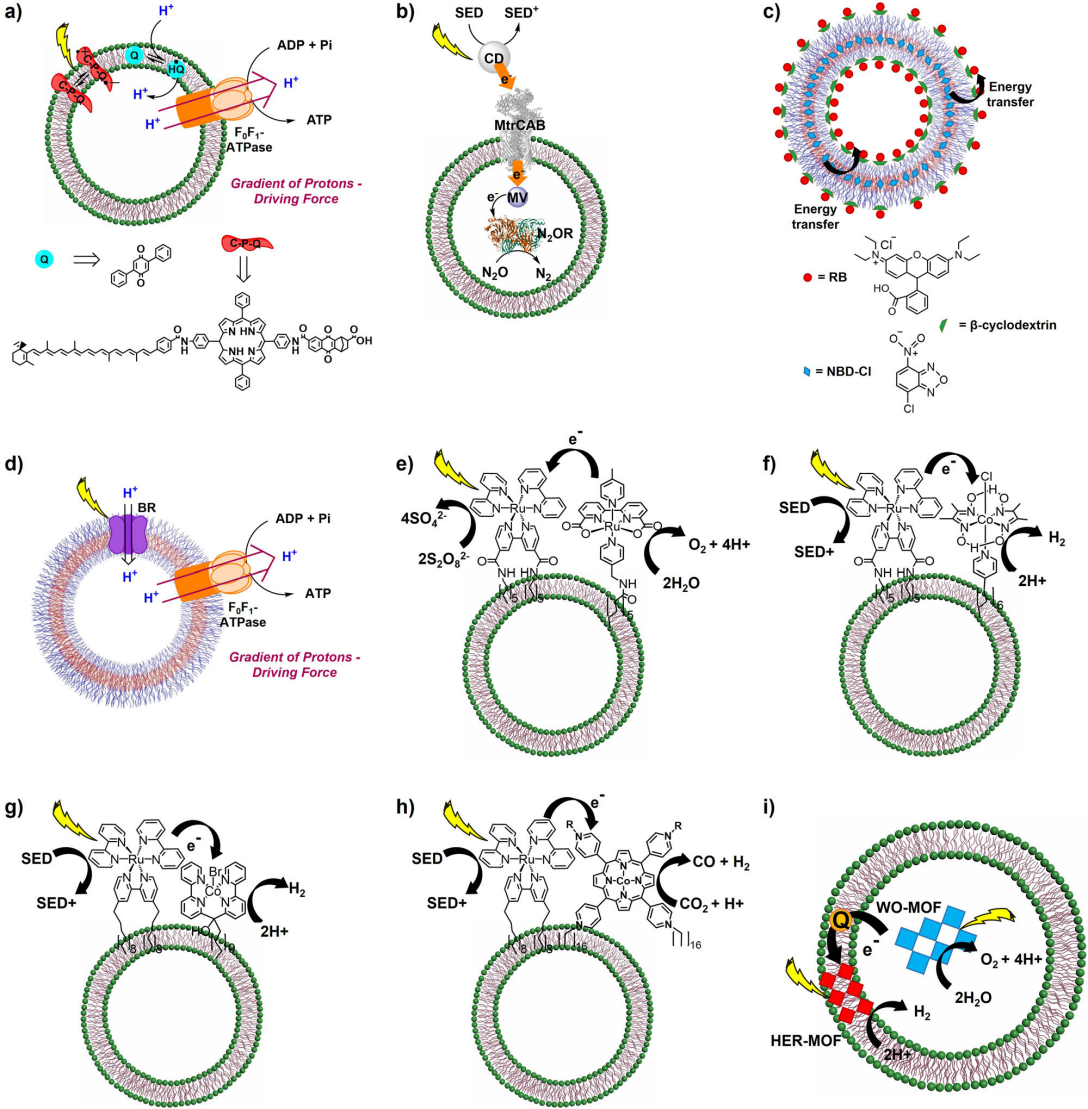
Selected examples of artificial vesicles for AP transformations. **a** Liposome embedding a synthetic molecular CPQ triad, quinone and a F_0_-F_1_-ATP synthase for electron and proton transfer across the membrane with concomitant pH gradient generation coupled to the synthesis of ATP reported by A. Moore et al.,^96^ (**b**) proteoliposome embedding MtrCAB from *S. oneidensis* MR-1 and encapsulated N_2_O reductase for electron transfer across membrane with N_2_O-to-N_2_ reduction reported by J. N. Butt et al.,^58^ (**c**) polymersome embedding donor and acceptor molecules at each domain of the membrane for transmembrane energy transfer reported by Y. Zheng, Y. Zhou et al.,^119^ (**d**) polymersome with embedded bacteriorhodopsin a F_0_-F_1_-ATP synthase for transmembrane proton transfer coupled to ATP synthesis reported by T. Vidaković-Koch et al.,^99^ (**e**) liposome containing a Ru-PS and a Ru-WOC for light-driven WO reported by L. Sun, B. König et al.,^127^ (**f**) liposome with a Ru-PS and a Co-HEC for light-driven HER reported by B. König et al.,^132^ (**g**) liposome with a Ru-PS and a Co-HEC for light-driven HER reported by S. Bonnet et al.,^130^ (**h**) liposome with a Ru-PS and a CoPc-CO_2_RC for light-driven CO_2_ reduction to CO reported by L. Hammarström, E. Reisner et al.,^93^ (**i**) liposome with a HER-MOF embedded in the hydrophobic membrane and an encapsulated WO-MOF for overall light-driven WS reported by W. Lin, C. Wang et al.^140^.

The use of simplified bioinspired artificial vesicles as model systems can shine a light on the intricate interplay of charge separation dynamics across membranes. Examples of bioinspired vesicular systems to study energy transfer, electron transfer, proton transfer, and transfer of other energy vectors across membranes have been reported in the last years^58–62,94–103^. A vast majority of those examples rely on liposomes, since their use gained increasing interest with the pioneer examples from the groups of JM. Lehn^94^, M. Calvin^21,104–106^ and F. Toda^107^ in the 70s, N. Toshihiko^108^ and J.H. Fendler^109^ in the 80s, and A. L. Moore and T. A. Moore^95,96^ in the 90s, showing that electron transfer across an artificial membrane was possible and that it could even be coupled to the generation of proton gradients. More concretely, Lehn and coworkers reported the light-induced electron transport across artificial membranes mediated by 2-methyl-1,4-naphthoquinone^94^. Later on, Moore et al. developed a model reaction center based on a synthetic carotene–porphyrin–naphthoquinone molecular triad (CPQ), where the electron donor and acceptor are linked to a photosensitive porphyrin group, as well as a quinone, which were incorporated into a liposome bilayer. Upon excitation, CPQ generates a reduction potential on the bilayer’s outer surface and an oxidation potential on its inner surface. This potential difference prompts the quinone to oscillate between its oxidized and reduced states, which is translated into proton transport across the membrane, with concomitant generation of a pH gradient^95^. Later on, they integrated the synthetic CPQ reaction center into liposomes containing a biological F_0_F_1_-ATP synthase, allowing the photogenerated proton gradient across the membrane to produce ATP (Fig. 3a)^96^. After these pioneer studies, F. Mavelli et al. reported the reconstitution of a photosynthetic reaction center from *R. sphaeroides* within a giant unilamellar vesicle for light-driven proton gradient creation through quinone reduction and protonation, inducing both pH and electrochemical potential gradients^110^. These initial studies demonstrated the potential of artificial vesicles to store light energy into the generation of proton gradients (chemical energy).

More recently, transmembrane electron transport in liposomes with reconstituted transmembrane cytochrome proteins was also reported by the groups of J. N. Butt, E. Reisner and L. J. C. Jeuken. The authors reported the integration of an icosa-haem transmembrane electron transfer protein (MtrCAB)^111,112^ isolated from *Shewanella oneidensis* MR-1 into a liposome membrane. And demonstrated electron transport across the membrane towards the inner compartment of the liposomes where either a redox-active dye, reactive red 120 (RR120)^62^, or a N_2_O reductase enzyme^58^ were encapsulated. In the first case, photoreduction of the embedded MtrCAB by an external synthetic light-harvester in the presence of an electron donor (EDTA) allowed for electron transport towards the inner space with concomitant bleaching of the RR120 band at 539 nm^62^. Likewise, the photoreduction of MtrCAB in proteoliposomes containing the N_2_O reductase (N_2_OR) enzyme allowed the reduction of the greenhouse gas N_2_O to N_2_ by the encapsulated enzyme upon electron transfer (Fig. 3b)^58^. More recently, the groups of M. Ohba^113^, A. Ikeda^114^ and S. Bonnet^115–118^ demonstrated efficient energy transfer across lipid membranes.

In the case of polymeric vesicles, the groups of Y. Zheng and Y. Zhou demonstrated that hyperbranched polymer-based polymersomes can be used as compartments for light-harvesting. They showed 80% efficiency of energy transfer between the spatially separated donor (4-chlor-7-nitro-2,1,3-benzodiazole, NBD-Cl), encapsulated in the inner compartment, and acceptor (rhodamine B, RB), anchored at the surface of the β-cyclodextrin-functionalized vesicles (Fig. 3c)^119^. In another example, Thordarsen et al. demonstrated efficient light-driven electron and proton transfer across the membrane of a polymersome based on polystyrene-polyacrylic acid block copolymer bilayer^120^. U. Schwaneberg and coworkers^121^, and the group of W. Meier^122,123^ showed efficient transmembrane proton transfer in polymersomes with a reconstituted OmpF channel protein. Other examples include light-driven electron transfer across polymeric membranes in protein-polymersomes, where photosynthetic natural reaction centres have been reconstituted into the membrane^57,124,125^.

Finally, polymeric vesicles have also been explored to study ATP synthesis in artificial compartments. The group of C. D. Montemagno demonstrated light-driven proton transfer across polymeric membranes with concomitant APT synthesis, when reconstituting bacteriorhodopsin and F_0_F_1_-ATP synthase into PEtOz-PDMS-PEtOz, [poly(2-ethyl-2-oxazoline)-b-poly (dimethylsiloxane)-b-poly(2-ethyl-2-oxazoline)], polymersomes^126^. Following a similar strategy, the group of T. Vidaković-Koch reported the light-driven ATP regeneration in hybrid polymeric vesicles made of poly(dimethylsiloxane)-graft-poly(ethylene oxide) (PDMS-g-PEO) graft copolymer and polybutadiene-b-poly(ethylene oxide) (PBd-PEO) diblock copolymer (Fig. 3d). The authors reconstituted bacteriorhodopsin (BR) and F_0_F_1_-ATP synthase in four different vesicles: (i) phosphatidylcholine (PC) liposomes, (ii) hybrid PC lipid/PDMS-g-PEO polymer vesicles, (iii) hybrid PC lipid/PBd-PEO vesicles, and (iv) hybrid PDMS-g-PEO polymer/ PBd-PEO polymer; demonstrating light-driven ATP production in all cases. Interestingly, despite a higher quantity of ATP was produced in PC liposomes (100%) and the hybrid lipid/polymer vesicles (98 and 92%, respectively) as compared to the PDMS-g-PEO/PBd-PEO ones (54%), PDMS-g-PEO/PBd-PEO vesicles showed enhanced functional durability (80% remaining activity after 42 days), thanks to the greater mechanical stability given by the polymeric membrane^99^. This work highlights the effect of the nature of the membrane scaffold in the stability of the overall photocatalytic activity. While in this study lipid-containing vesicles are more active for ATP production, they are less stable. Substitution of the liposome by a polymersome in this case enhances the stability of the overall system. This finding is promising to develop compartmentalized systems with enhanced durability to allow for mechanistic studies towards rational system design. Therefore, the choice of the membrane is pivotal in the design of compartmentalized systems for a target chemical transformation.

## Compartmentalized photocatalytic systems for WO

The interest in using artificial vesicles for compartmentalized AP reactivity has led to the exploration of vesicular systems for WO^9,10,127–129^. The first example of a self-assembled dual photocatalytic system for WO was reported by the group of L. Sun and B. König. They reported the modification of the state-of-the-art ruthenium tris(bipyridine)ruthenium(II) photosensitizer (PS) [Ru(bpy)_3_]^2+^, and the [Ru(bda)(pic)_2_] (bda = 2,2′-bipyridine-6,6′-dicarboxylato, pic = 4-methylpyridine) and [RuL(pic)_3_] (H_2_L = 2,6-pyridinedicarboxylic acid) WO catalysts (WOCs) with hydrophobic alkyl chains to embed them into a phospholipid membrane for compartmentalized solar-driven WO using sodium persulfate as sacrificial electron scavenger (Fig. 3e). The biomimetic vesicles allowed efficient WO even at low catalyst concentrations (500 nM), not possible in homogeneous conditions. This was rationalized by the increase in the local concentration of the redox partners and the shorter average distance for electron transfer between them^127^. Following a similar strategy, the group of S. Bonnet reported a detailed mechanistic study of liposomes for light-driven WO upon the anchoring of [Ru(bpy)_3_]^2+^ together with [Ru(bda)(pic)_2_] modified with hydrophobic alkyl chains to the lipid bilayer. Upon kinetic studies, the authors showed an increased efficiency of the reduction of the photooxidized Ru-PS by electron transfer from the Ru-WOC, which is the rate-determining step (RDS) in homogeneous conditions, and observed that in the liposomes the oxidative quenching of the excited Ru-PS by the sacrificial electron scavenger was RDS, which increased the overall stability of the system^128^.

## Compartmentalized photocatalytic systems for HER and CO_2_R

The potential of artificial vesicles to develop compartmentalized systems for reductive transformations has also garnered substantial attention. In this context, liposome- and other membrane-based systems for photocatalytic HER^130–133^, CO_2_R^93,134–136^, and organic transformations^58,103^, have been reported.

In the case of hydrogen evolution, pioneer work was reported by the group of B. König where they developed self-assembled functionalized membranes for photocatalytic HER by co-embedding the [Ru(bpy)_3_]^2+^ PS together with a Co hydrogen evolution catalyst (HEC) [Co(dmgH)_2_(py)Cl] (dmgH = dimethylglyoximate) modified with hydrophobic chains in phospholipid membranes. As observed with other vesicular systems, the self-assembly allows the proximity between the PS and the HEC, resulting in high turnover number (TON) H_2_, up to 165, in optimized aqueous conditions.

This improved photocatalytic activity in fluid membranes suggests that dynamic reorganization within the membrane enhances visible-light-driven HER^132^. With a similar strategy, L. Sun, B. König et al., reported self-assembled liposome vesicles with a modified hydrophobic [Ru(bpy)_3_]^2+^ PS embedded in the membrane, and a hydrophilic [FeFe]-H_2_ase subunit mimic adsorbed on top of the membrane for light-driven HER. As in the previous case, the arrangement of complexes along the membrane allows close proximity between them, resulting in a 6- to 12-fold increase in the TON H_2_ as compared to the same non-immobilized system in solution^131^. In another example, S. Bonnet et al. developed 1,2-dimyristoyl-sn-glycero-3-phosphocholine (DMPC) and 1,2-distearoyl-sn-glycero-3-phosphoethanolamine-N-[methoxy(polyethylene glycol)-2000] (NaDSPE-PEG2K) based liposomes containing the modified hydrophobic [Ru(bpy)_3_]^2+^ PS together with an alkylated Co(II) polypyridyl HEC for photocatalytic HER. Mechanistic studies showed that the activity was limited to the decomposition of the Ru-PS^130^.

For CO_2_R, the first example was reported by the group of S. Murata. They developed a liposome with a [Ru(dtb)(bpy)_2_]^2+^ PS and Re(dtb)(CO)_3_Cl complex (dtb = 4,4’-ditridecyl-2,2’-bipyridyl) incorporated in the membrane for light-driven CO_2_R to CO in aqueous solution under visible light and using ascorbic acid as sacrificial electron donor, obtaining 190 TON CO. In this case, the assembly allows the use of the water-insoluble Re(dtb)(CO)_3_Cl complex to be used in aqueous solutions^134^. Later on, S. Bonnet and colleagues studied a similar system but varying the lengths of the alkyl chains to embed the Ru and Re complexes into the liposomes. The authors demonstrated that the use of shorter alkyl chains was translated into a higher mobility of the molecules on the lipid membrane, and thus faster electron transfer between the Ru and Re complexes^135^. Using a similar strategy, the groups of S. Bonnet, L. Hammarström and E. Reisner reported the assembly of DMPC and NaDSPE-PEG2K liposomes containing a [Ru(bpy)_3_]^2+^ PS together with a 5,10,15,20-(tetra-N-hexadecyl-4-pyridinium)-porphyrin Co(II) (CoPc) complex modified with alkyl chains for photocatalytic CO_2_R using ascorbic acid as sacrificial electron donor under visible light irradiation. The system achieved almost a 5-fold higher activity (1456 TON CO) and 7-fold selectivity (77%) for CO_2_R to CO compared to the homogeneous non-alkylated analogous system, revealing an enhanced performance thanks to the self-assembly. Mechanistic studies demonstrated that the grater performance is due to a 2-fold increased charge-separation state lifetime of the PS, allowing for a 9-fold faster electron transfer to the CoPc in the liposome^93^. More recently, S. Takizawa et al. reported DPPC liposomes that contained a cationic and an anionic Ir PSs ([Ir_1_^+^] and [Ir_2_^-^], respectively) together with a Re(dtb)(CO)_3_Cl catalyst embedded in the lipid bilayer membrane for light-driven CO_2_R to CO. The key of this system is the transference of triplet excitation energy from [Ir_2_^-^] to [Ir_1_^+^] due to Coulombic interactions to generate the triplet excited state of [Ir_1_^+^], which then reduces Re(dtb)(CO)_3_Cl to form the active catalytic species^137^. Finally, more complex compartmentalized systems have been reported for CO_2_ conversion, including enzymes and full biologic metabolic pathways into artificial vesicles^136,138,139^.

## Biomimetic vesicular systems for full AP

Reported examples of compartmentalized systems for overall AP remain scarce due to the intrinsic challenge it represents to combine oxidation and reduction transformations (with their specific oxidation and reduction reaction centres) within a vesicle. To the best of our knowledge, there is only one example where a liposome is used for overall photocatalytic WS, using the electrons derived from WO to evolve H_2_. This system was reported by W. Lin, C. Wang et al. and consists of integrated HER- and WO-MOF nanosheets into liposomes to separate both reactions while allowing their synergy for overall WS. The HER-MOF nanosheets are constructed by Zn–porphyrin (PS) and Pt–porphyrin (HEC) moieties functionalized with hydrophobic groups to facilitate their incorporation into the lipid bilayer. The WO-MOF consists of a [Ru(bpy)_3_]^2+^ PS and an Ir–bipyridine WOC located in the hydrophilic interior of the liposome membrane. This system achieved overall photocatalytic WS with 1.5 ± 1% quantum yield due to efficient charge separation in the lipid bilayer, and an ultrafast electron transfer from the Zn–porphyrin and [Ru(bpy)_3_]^2+^ to the HER and WO reaction centres in the MOFs thanks to the liposome membrane^140^. However, to the best of our knowledge, there are no examples of polymersomes for compartmentalized WO, WR or CO_2_ separately, nor of any kind of artificial vesicle (based on polymers or lipids) for overall WO-to-CO_2_R towards solar fuel and chemical production so far^9,67,75,141^.

## Outlook

### Current state-of-the-art

The importance and ubiquity of membranes in biological transformations, and the need to better understand their function in different biochemical processes have promoted the development of artificial vesicles for chemical transformations. In the recent years artificial vesicles have attracted special attention to develop photocatalytic systems for AP, with the goal to mimic the structural arrangement and confinement of the thylakoid membrane in chloroplasts. However, from the previously reported studies summarized in this review, we infer that developing such photocatalytic systems for compartmentalized AP is very challenging due to the different aspects that need to be considered: (i) choice of the membrane scaffold and its properties, (ii) careful selection of catalysts, photosensitizers and mediators, (iii) separation of reaction centres at each side of the membrane, (iv) efficient charge separation, (v) ensuring a unidirectional electron transfer, (vi) product selectivity, (vii) concentration of catalysts, photosensitizers and mediators within the membrane, among others. A tight control and rational design of these parameters is pivotal for the overall stability and performance of the photocatalytic systems. For instance, a common feature of the previously reported studies is that immobilization of catalysts and photosensitizers onto biomimetic membranes increases their stability, and the proximity between reaction components, which can lead to an enhancement in the electron transfer rates between them. Given the complexity outlined in the development of artificial vesicles for compartmentalized AP, it’s unsurprising that, despite the advancements in energy, proton, and electron transfer studies within vesicular systems, as well as progress in light-driven WO, CO_2_R, HER, and WS, a fully integrated vesicle-based system that spans the complete WO-to-CO_2_R process has not yet been achieved. Therefore, the development and study of these systems are necessary to establish the foundations for their advancement in the field of compartmentalized production of solar fuels and chemicals. While the application of such systems for large scale fuel and chemical production is uncertain, they enable the exploration of coupling two different half-reactions (i.e. WO and CO_2_R) within a single system and offer a platform for devising innovative solutions to mitigate charge recombination and back reactions, addressing the primary challenge in photocatalytic AP. Moreover, these systems can act as simplified models to study processes endemic to membranes, such as transmembrane proton and electron transfer, which are often less accessible in native biological systems. Additionally, they can serve as model platforms to investigate charge separation dynamics at interfaces, with implications in other areas, such as the study of charge dynamics in semiconductor materials.

### Future directions

Future research should aim at the realization of full WO-to-CO_2_R photocatalytic vesicles. To this end, progress in different areas is required. First, the development of more stable and chemically versatile vesicles that can mimic the thylakoid membrane more effectively. Second, further development of vesicles with tuneable properties like permeability control and stimuli response. These strategies might include the development of dynamic compartments that can adapt their structural and functional properties in response to environmental stimuli, which will be important for derived photocatalytic product harvesting or to develop complex cascade reaction systems within vesicles. Moreover, improvement of the overall photocatalytic efficiency and unidirectional electron transport requires optimized spatial arrangement, specific orientation and controlled proximity of reaction partners at each side of the bilayer membrane. This might benefit from the rational modification of catalysts and photocatalyst to have specific binding sites for their covalent attachment to the membrane (in their respective complementary binding sites). Regulation of the internal environment of the compartments is another area that requires attention because can affect the photocatalytic activity and selectivity. Future systems could incorporate sensing mechanisms that allow for the real-time control of internal pH, ion concentration, and other critical factors for optimizing reaction conditions. Finally, given the complexity and multifaceted challenges of compartmentalized AP systems, there is a need for increased collaboration across disciplines to advance in the field.
